# Infection control: point prevalence study versus incidence study in Polish long-term care facilities in 2009–2010 in the Małopolska Region

**DOI:** 10.1007/s15010-012-0351-5

**Published:** 2012-10-20

**Authors:** J. Wójkowska-Mach, B. Gryglewska, J. Czekaj, P. Adamski, T. Grodzicki, P. B. Heczko

**Affiliations:** 1 Chair of Microbiology, Jagiellonian University Medical College, Krakow, Poland; 2 Department of Internal Medicine and Gerontology, Jagiellonian University Medical College, Krakow, Poland; 3 Nursing Home, Krakow, Poland; 4 Institute of Nature Conservation, Polish Academy of Sciences, Krakow, Poland

**Keywords:** Long-term care facilities, Point prevalence study, Incidence study, Target surveillance

## Abstract

**Objective:**

The objective of this study was to evaluate the epidemiology of infection in Polish long-term care facilities (LTCFs) and to analyse the capabilities and legitimacy of implementing continuous targeted surveillance.

**Methods:**

The study investigated the relationship between the presence of infection and health status, tested using a point prevalence study (PPS) and incidence study. A 1-day PPS was carried out in October 2009, with prospective continuous surveillance between December 2009 and November 2010. Infections were defined according to McGeer’s criteria.

**Results:**

The surveillance encompassed 193 people. The prevalence was 14.0 % in residential homes (RHs) and 18.7 % in the nursing home (NH). Various types of infections (in the PPS) were observed significantly more frequently in patients with asthma, wounds, atherosclerosis of lower extremities, tracheotomy tubes and conditions in patients hospitalised in intensive care units (ICUs) up to 1 year before the PPS day. The incidence rate was 2.7/1,000 patient days (pds).

**Conclusions:**

The factors determined to be important for the risk of infection (in the continuous study) include the general status of patients, expressed using Barthel, abbreviated mental and Katz scales, as well as limited physical activity, stool incontinence and urinary catheterisation. In the PPS study, only a slight relationship was shown between the general status of residents and the risk of infection. None of the general status scales used clinically were shown to be helpful in estimating that risk, similarly to the five-point physical activity scale. Prospective continuous surveillance shows a possibility of limiting the range of infection control in the LTCFs within targeted surveillance in a population of patients that requires intensive nursing procedures. As a marker, one could point to the low score in the Barthel or Katz scales or low physical activity/bedridden persons.

## Introduction

In developed countries, the population of elderly people is steadily increasing, which is associated with increased needs for permanent institutional care. In 2009 in Poland, 13.3 % of people were aged 65 years or older, and the annual growth for this age group is about 57,000 people [1]. Approximately 17,500 persons in this group need permanent care in different types of nursing homes and 53 % of them are bedridden. The demographic structure of people in this age group is highly variable. In 1980, people over 65 years of age comprised 10.1 % of the total population, 13.5 % in 2009 and, according to demographic projections, in the year 2035, it will be 23.2 %. The increase in the proportion of the oldest group in the general population of Poles, i.e. aged 85 years and above, increased from 0.7 % in 1989 to 1.3 % in 2009 [2]. The increasing population of people aged over 65 years makes the health problems of this age group dominant in the health care system not only in Poland but throughout the world. With age, there are an increasing number of persons demanding long-term care, whose primary role is to provide an older person with the longest possible operational independence. Home care in the older person’s place of residence applies in most of the geriatric population with some degree of disability. However, with much infirmity, especially in combination with a number of chronic diseases, it often needs to become institutionalised. It is estimated that between 10 and 25 % of older people may require placement in various forms of care homes [3]. On the other hand, changes in the immune system occurring during the aging process pose a higher risk of infections in this population [4]. The significance of these changes for healthy elderly people is limited, but with many chronic conditions and polypharmacy, malnutrition occurs secondary to the deterioration of the immune function [5]. Residents of nursing homes are at a particularly high risk, among which a significant degree of functional disability with urinary incontinence and bowel incontinence, cognitive impairment, weak cough reflex, dysphagia and reduced mobility are often present. The recognition of infection in this population is difficult due to the increased likelihood of atypical infection presentation [6, 7].

At present, infections have never been evaluated in Polish long-term care facilities (LTCFs). Hence, the objective of this study was to estimate the epidemiology of infection, depending on the type of LTCF. The second aim was to assess the relationship between the frequency of infection and health status, tested using a point prevalence study (PPS) and incidence study for the analysis of capabilities to implement targeted continuing surveillance only in the high-risk portion of LTCF residents.

## Materials and methods

### Part I: point prevalence study (PPS)

A 1-day PPS was carried out in October 2009 in three LTCFs: two residential homes (RHs) and one nursing home (NH). All institutions implemented a voluntary protocol and cooperated with an external researcher. A resident was defined as a person being a resident in LTCFs for >48 h during the study day. An NH was defined as an institution where residents need medical or skilled nurses’ supervision 24 h a day and which provides more intensive health care than an RH, where residents are unable to live independently and require supervision or assistance with the activities of daily living. Infections were defined according to McGeer’s criteria [8] and were detected by trained health personnel with the cooperation of the project worker.

Information on potential risk factors was collected from the medical history, medical and nursing records, and recorded on a questionnaire form. The functional status of the study participants was estimated using the Katz scale (scores range from 0 to 6) and Barthel’s index (scores range from 0 to 100) [9]. Physical dependence was also classified according to the following five-point scale: *1* independent, *2* independent with falls, *3* limitations in movement, *4* bedridden, mobile, *5* bedridden, dependent. The data collection protocol had been previously tested and revised in a pilot study. Material for microbiological examination was collected, depending on the clinical status of patients, i.e. wound swabs, pharyngeal swabs, sputum, urine and others.

### Part II: continuing surveillance

Prospective continuous surveillance was carried out from December 2009 to November 2010 in the same three LTCFs. Infections were defined according to McGeer’s criteria [8] and were detected by trained health personnel. Amongst the patients enrolled in the study, two persons dropped out and 31 patients died.

The relation between types of care, socio-demographic characteristics, and incidence and prevalence of infections were analysed with two main groups of statistical techniques: if the numerical parameters (age, length of stage etc.) were compared by a nominal characteristic (type of care, form of infection etc.), a simple analysis of variance (ANOVA) test was used. If the distribution of numerical characteristics did not fit the normal distribution, the non-parametric alternative of ANOVA, the Kruskal–Wallis test, was used. For the contingency of nominal characteristics, Chi-square (χ^2^) and likelihood ratio frequency tests were used. In cases where the categorical variable (i.e. type of care) was obviously dependent on the parameters measured in a continuous scale (age, Barthel’s scale), the significance of such relations was analysed using logistic regression.

To obtain the sensitivity of the proposed inclusion criteria based on Barthel’s scale, receiver operating characteristic (ROC) curve analysis was used. As this analysis demands the criteria of “true-positive” and “false-positive” recognition, they were based on the following assumptions: residents with endemic infections fulfilling at least one of the following criteria were included in the group that required the monitoring of: urinary catheter, tracheostomy tube, feeding via gastric catheter, stool incontinence or stroke. The common results of several risk factors on the probability of infection were analysed with multivariable models based on nominal logistic regression.

*p*-values of <0.05 were considered to be statistically significant. All analyses were performed using the SAS JMP 7.01 and R 7.0.2 packages.

The study was approved by the Ethics Committee at the Jagiellonian University and it conforms to the guidelines set forth by the Declaration of Helsinki.

## Results

### Description of population

Surveillance (PPS and incidence study) encompassed 193 people, of which 86 comprised residents (44.6 %) belonging to the RHs group and 107 persons (55.4 %) made up the NH group. The total number corresponded to 2.6 % of the total population of NH residents (*n* = 4,154) in the Małopolska Region for the year 2009 [10].

The study population was not a homogeneous population; it differed significantly in body weight, general condition expressed as various (chronic) diseases, problems maintaining personal hygiene expressed as urinary and/or stool incontinence, use of tracheotomy tubes, enteral feeding and frequency of hospitalisation before the PPS day (Table 1). Differences were observed in the general condition of residents, expressed specifically in the Barthel or Katz scales—that is, those with a low scale with a low value were significantly more likely to be under NH care—and physical activity, in that NH residents had significantly more limited mobility. Invasive procedures such as feeding by gavages, tracheotomy or bladder catheterisation—at least one—was performed in 36 % of the test group, with no intravascular injections being applied (Table 1).

**Table 1:** Characteristics of the studied population at baseline

	RHs (no./%)		NH (no./%)		*p* value
Male residents	36	41.9	35	32.7	–
Residents with recent hospitalisation	20	23.3	8	7.5	<0.001
Intensive care unit	2	2.3	1	0.9	
Internal medicine unit	13	15.1	4	3.7	
Surgery unit	5	5.8	1	0.9	
Normal body weight	75	87.2	82	76.6	0.003
Overweight	9	10.5	16	15.0	
Obesity	0	0.0	0	0.0	
Malnutrition/cachexia	2	2.3	6	5.6	
Diabetes	23	26.7	31	29.0	–
Asthma/chronic obstructive pulmonary disease	17	19.8	19	17.8	–
Venous insufficiency	21	24.4	15	14.0	–
Pressure sore	3	3.5	6	5.6	–
Prostatic hyperplasia	10	27.8	19	54.3	0.010
Stroke	5	5.8	25	23.4	<0.001
Urinary incontinence: diapers	23	26.7	39	36.4	<0.001
Bladder catheterisation (on a permanent basis)	0	0.0	39	36.4	
Stool incontinence	6	7.0	67	62.6	<0.001
Peripheral vascular disturbances	24	27.9	32	29.9	–
Atherosclerosis of lower extremities	8	9.3	14	13.1	–
Varicose veins of lower extremities	19	22.1	12	11.2	0.020
Dysphagia	4	4.7	26	24.3	<0.001
Gastric catheter	0	0.0	26	24.3	<0.001
Tracheostomy tube	1	1.2	4	3.7	–
Gastrostomy tube	0	0.0	3	2.8	–
Ulcers	5	5.8	4	3.7	–
Other wounds	1	1.2	1	0.9	–
Feeding via gastric catheter	0	0.0	24	22.4	<0.001
Feeding via gastrostomy tube	0	0.0	3	2.8	–
Age, mean ± SD (95 % CI) (years)	76.2 ± 10.5 (73.9–78.4)		76.8 ± 11.1 (74.6–78.9)		–
Length of stay in the facility, mean (years)	6.5		4.2		–
Barthel’s index, mean ± SD (95 % CI)	75.6 ± 34.5 (68.2–83.0)		19.5 ± 17.4 (16.1–22.9)		0.001
Katz scale, mean ± SD (95 % CI)	4.7 ± 2.1 (4.2–5.1)		1.3 ± 1.6 (0.9–1.6)		<0.001
Physical activity^a^, mean ± SD (95 % CI)	1.9 ± 1.2 (1.7–2.2)		3.7 ± 1.1 (3.5–3.9)		<0.001
Estimated number of infections in 2008, mean	1.4		0.6		<0.001

*RHs* residential homes, *NH* nursing home, *SD* standard deviation, *CI* confidence interval

^a^Physical activity, expressed on a five-point scale: *1* independent patient, no limitations, *2* independent no limitations, recurrent falls, *3* limitations in mobility, *4* bedridden, able to change body position on his/her own, *5* bedridden, dependent on others

### Part I: PPS

Thirty-two cases of infection were detected in 30 persons, and the prevalence was 14.0 % in RHs and 18.7 % in the NH. Various types of infections were observed significantly more frequently in patients with asthma, various types of wounds (pressure sore, ulcers and others), atherosclerosis of lower extremities and tracheotomy tubes and conditions in patients hospitalised in the intensive care unit (ICU) within 1 year before the PPS day. There was no significant association between prevalence and diabetes, venous insufficiency, stroke, prostatic hyperplasia, the value of the Barthel or Katz scales and other determinants of health deficits, such as urinary and/or stool incontinence or dementia. In addition, age did not influence the risk of developing an infection. Among the infections, infections of the skin and the upper respiratory tract were dominant (Table 2).

**Table 2:** Characteristics of patients with at least one detected infection in the point prevalence study (PPS) and in the prospective continuing surveillance (CS) study (*n* = 193)

	Participants with an infection(s) in the PPS (no./%)		*p* value	Participants with an infection(s) in the CS (no./%)		*p* value
Male residents	11	14.3	–	34	44.2	–
Type of care						
Residential home	12	14.0	<0.001	40	46.5	–
Nursing home	20	18.7		53	49.5	
Asthma/chronic obstructive pulmonary disease	14	7.3	0.020	23	11.9	–
Pressure sore	11	5.7	<0.001	9	4.7	–
Urinary incontinence: diapers	15	7.8	–	37	19.2	0.034
Urinary catheterisation (on permanent basis)	10	5.2		24	19.2	
Stool incontinence	17	8.8	–	45	23.3	0.021
Atherosclerosis of lower extremities	12	6.2	0.004	19	9.8	–
Tracheostomy tube	1	0.5	0.001	5	2.6	0.006
Ulcers	4	2.1	–	8	4.1	0.011
Other wounds	3	1.6	0.007	3	1.6	–
Feeding via gastrostomy tube	1	0.5	–	3	1.6	0.034
Age mean ± SD (95 % CI) (years)	79.5 ± 12.0 (75.9–83.1)		–	78.0 ± 11.4 (75.6–80.2)		–
Barthel’s index, mean ± SD (95 % CI)	48.2 ± 39.3 (36.2–60.1)		–	41.1 ± 39.2 (33.4–48.8)		0.009
Katz scale, mean ± SD (95 % CI)	2.8 ± 2.6 (2.1–3.6)		–	2.5 ± 2.5 (2.0–3.0)		0.006
Physical activity^a^, mean ± SD (95 % CI)	3.1 ± 1.6 (2.6–3.6)		–	3.1 ± 1.6 (0.1–3.1)		0.041

*RHs* residential homes, *NH* nursing home, *SD* standard deviation, *CI* confidence interval

^a^Physical activity, expressed on a five-point scale: *1* independent patient, no limitations, *2* independent no limitations, recurrent falls, *3* limitations in mobility, *4* bedridden, able to change body position on his/her own, *5* bedridden, dependent on others

Biological materials for bacteriological examination were collected from all 32 symptomatic infections, and microbiological confirmation was obtained in two cases out of 11 lower respiratory tract infections. The dominant aetiological factors of microbiologically confirmed infections were *Staphylococcus aureus* (19.4 %) and Gram-negative bacilli: *Escherichia coli* and *Pseudomonas aeruginosa* (Table 3).

**Table 3:** Cases of infections and their aetiology in the PPS versus incidence study

Clinical type of infection	Point prevalence study (PPS)			Incidence study			
	No.	%	Prevalence rate (%)	No.	%	Cumulative rate (%)	Density rate (1,000 pds)
Wound	17	53.1	8.8	19	10.9	9.8	0.3
Upper respiratory tract	11	34.4	5.7	2	1.4	1.0	0.0
Lower respiratory tract	3	9.4	1.6	11	6.3	5.7	0.2
Pneumonia	0	0.0	0.0	42	24.0	21.8	0.7
Urinary tract infection	1	3.1	0.5	30	17.1	15.5	0.5
Others	0	0.0	0.0	71	40.6	36.8	1.1
Total	32	100.0	16.6	175	100	90.7	2.8

Microbial etiology	No	%	No	%			
*Staphylococcus aureus*	7	19.4	8	9.5			
*Pseudomonas aeruginosa*	7	19.4	25	29.8			
*Escherichia coli*	7	19.4	11	13.1			
*Klebsiella* spp.	4	11.1	10	11.9			
*Proteus* spp.	3	8.3	10	11.9			
*Acinetobacter* spp.	2	5.6	3	3.6			
Other	6	16.7	17	20.2			
Total	36	100.0	84	100.0			
Polyaetiological	4	22.2	17	32.1			

### Part II: continuing surveillance

The study was performed in the years 2009 and 2010. The total number of patient days amounted to 62,035 in LTCFs. The study involved 193 residents in three LTCFs; 94 residents (48.2 %) had symptoms of infection, while the other participants did not show signs of any infections. In the studied group of 94 persons (48.2 % of the total), 175 cases of infections were detected. These infections were most commonly respiratory tract infections and pneumonia, as well as urinary tract infections. The total incidence was 2.7/1,000 patient days (pds); it was higher in the NH at 3.6/1,000 pds compared to 1.9 [relative risk (RR) 1.9] in RHs, but these differences were not significant. One gastrointestinal (GI) tract infectious outbreak associated with norovirus aetiology was found among 25 residents of RHs. There were 157 cases of infections other than GI (endemic) infections observed in 75 residents.

Factors important for the risk of any infection were the general status of patients, expressed using the Barthel and Katz scales, as well as limited physical activity, stool incontinence, urinary catheterisation, tracheostomy tube, ulcers in PPS and feeding via gastrostomy tube (Table 3). No correlation was found with diabetes, venous insufficiency, stroke or prostatic hyperplasia.

The multivariable analysis of the common influence of age, physical activity, Katz scale and Barthel score (*c* ^2^ = 12.1362; *df* = 5; *p* = 0.0330) resulted in a rather low predictability (*R* ^2^ = 0.0475). ROC curve analysis of sensitivity supports these conclusions, as the calculated sensitivity of the model is only 0.64251. The model that also includes a categorical variable is slightly more significant (χ^2^ = 28.8014; *df* = 16; *p* = 0.0253) and more predictable (*R* ^2^ = 0.1124). The parameter analysis shows that significant influence was confirmed for two factors: tracheotomy tube (*p* = 0.380) and ulcers (*p* = 0.0280). Close to significance was also found for asthma (*p* = 0.701). The ROC curve analysis shows that the sensitivity of the model is equal to 0.69242.

In the group of residents described by the Barthel scale as 15 or less (a total of 67 persons—34.7 % of the population), there were 81 cases of endemic infections observed in 42 patients (62.7 % of such residents), which accounted for 51.6 % of all such infections. The incidence of endemic infection was significantly dependent on the physical condition of the residents, as expressed by the Barthel scale. The lowest value of the scale, 0, was associated with a 62.5 % risk of developing this type of infection (RR 1.8), as well as in residents with a scale value of 1–15 (incidence 62.9 %, RR 1.9). In residents with higher values of the scale, described as 16–50, 51–75 and 76–100, significantly lower morbidity rates were observed, respectively: 40.0 % (RR 1.0), 18.2 (RR 0.5) and 15.0 % (RR 0.3).

Based on the assumed Barthel scale, the sensitivity value for including these patients in two critical groups (0 and 1–15 were calculated) was 0.9355 (*R* ^2^ = 0.9616, area under the curve = 0.824).

During the study period (1 year), changes in the condition of residents were observed in only 24 cases. The average time between any change was 190 days (SD 106).

Thirty-one persons died during the study (mortality 16.1 %) and 16 deaths (51.6 %) were directly related to the developed infections.

Fifty-three biological samples in total were collected for microbiological examinations (30.3 % of all cases of infections). Materials for microbiological examination were collected from 14 cases of wound infection, seven cases of pneumonia and seven cases of urinary tract infections and others.

The dominant aetiological agents were *P. aeruginosa* (29.8 %) (Table 3).

## Discussion

The results presented originate from the first infection surveillance system in Polish LTCFs.

The study encompassed various populations of patients and was conducted in two parts: PPS and continuing surveillance study (incidence study). The rates from the two studies cannot be compared directly, owing to different methodologies, but both are used in national programmes in Europe and throughout the world. In Italy, the incidence was estimated to be 11.8/1,000 pds, with a prevalence of 8.4/100 residents [11, 12], and in Norway, it was 5.2/1,000 pds and 6.7/100 residents, respectively. In the USA, incidence was shown to be 7.7/1,000 pds [13], in Canada 1.8–9.5/1,000 pds [14] and in Germany 6/1,000 pds [15]. The methodology of the two types of studies is different, and the results are incomparable, but the results are different than what was expected. The prevalence rate was much higher than expected in both studied LTCF populations (RHs and NH), but incidence rates were lower than expected for both populations. However, infections are common among residents in LTCF, with a frequency comparable to rates observed in acute care facilities [11, 12]. The prevalence of infections depends on the profile of the residents of LTCFs—they are very heterogeneous populations. Patients vary in age, may be admitted for psychiatric or medical care and, moreover, may remain in LTCFs permanently or for a given period. The frequency of infections is higher among elderly residents who permanently stay in an LTCF. Our study only involved such a population.

The reason for the observed differences (between high prevalence and low incidence) was also the method of data collection, as the PPS was performed in cooperation with an external investigator, who based his findings mostly on ward documentation and, secondly, on nurse and doctor interviews, while the continuing study was performed mainly by the physician in charge in cooperation with specially trained nursing personnel, which may have resulted in differences in sensitivity. However, we are not able to exclude the effects of some prevention strategies, because almost all residents of the NH had undergone vaccination against influenza and pneumococcal disease. Infections in LTCFs are usually considered to be endemic infections, outbreaks and/or the presence of alert pathogens with multiple antimicrobial resistances. The most frequent endemic infections are respiratory tract infections, urinary tract infections, and skin and soft tissue infections [11]. However, skin infections were the most frequent in our residents. This was probably connected with the clinical status of those persons, because they suffered from atherosclerosis of lower extremity arteries, had mobility problems and, consequently, skin ulcers and pressure sores.

Among the risk factors of infections, advanced age is usually an important factor increasing this risk. In our observations, however, age did not increase the incidence of disease. Only an insignificant trend of more pronounced and frequent development of infections appeared in the subgroup of the oldest residents (>90 years of age). The average age of patients with and without infections did not differ significantly (about 78 vs. 76.6 years), but it was lower than the age of residents of such facilities in other countries.

Nevertheless, the results show that there is a need for improvement not only when it comes to the sensitivity of the surveillance but also high prevalence rates, i.e. the risk of infection for a resident of a Polish LTCF is high.

There are significant differences in the identified types of infections between prevalence and incidence. The main reason is methodology: the short time frame of the PPS is too sensitive for, for example, pneumonia, because residents with pneumonia are more likely to be hospitalised than patients with various forms of wound infections usually treated in an LTCF. For the surveillance of rapidly progressing disease, like lower respiratory tract infections or urinary tract infection, a continuous study is more appropriate. Thus, even though the presence of the test is easier and less expensive, it does not include all phenomena which are infections in LTCFs.

The presented analysis also points to various results when it comes to the risk factors for developing an infection. The PPS results showed only a slight relationship between the general status of patients and the risk of developing an infection. None of the commonly used scales for estimating the patients’ general status, e.g. the Barthel or Katz scales, were helpful in estimating the risk of developing an infection, similarly to physical activity described in a five-point scale. This shows the arbitrary way that these infections occurred. The incidence study, on the other hand, showed more expected results, with confirmation of the relationship between many procedures characteristic for LTCFs. In this study, the data on the general status of patients was also confirmed as having value in the surveillance of residents. The results shown should be helpful in performing infection surveillance in LTCFs.

The prospective active surveillance for endemic infections in LTCFs should be restricted to the selected groups of residents with the highest risk of infection (low score on the Barthel or other scales). Targeted incidence surveillance could be a valid alternative: a Barthel score of 15 was showed to have the best sensitivity and specificity and, together with other risk factors, proved to have a good predictive value—thus, patients with a Barthel score of 15 or more could be included in such a targeted surveillance. Such targeted surveillance is particularly important in countries such as Poland, with limited resources in infection control, which is generally associated with both a small number of infection control officers and a lack of infection surveillance infrastructures in the LTCF. A focus on the high-risk population might be a solution for cost-effective surveillance; similarly among hospitalised patients [16], it seems that this type of approach could also be implemented in the LTCF.

During the study period, the average time between any change of health status of the residents was 190 days (SD 106). However, as the lower limit of the 95 % confidence interval for the calculated mean is 149, we propose this value as the check value for the Barthel scale period. For practical reasons, it is reliable to check it every 6 months.

Both prevalence and incidence methods of surveillance are currently recommended by the EU [17], but, at the same time, beginning in 2006, there has been an on-going European programme called Improving Patient Safety in Europe (IPSE) [18]. It seems reasonable not to limit the studies of infections in LTCFs only to PPS, but to wage the decision of the need to introduce prospective continuous surveillance based on the specific situation.

This has a special meaning in countries that are only preparing to introduce surveillance to LTCFs, such as Poland, where the development in this field of health care is not complete. A good confirmation of this is the age of residents encompassed in this study, which was much lower than the age of LTCF residents in other countries. In Norway, 78 % of residents were >81 years old [19], in Italy, the median age was 81 years [12], with values of 89 years in Canada [14] and 83 years in Germany [15]. That may be the reason for the lower incidence of infections than that seen in the literature and important information for public health.

A main source of the study limitation is the small number of patients included. This was due to a mistrust of residents (who had to agree to participate) and staff who participated in such studies for the first time, as they had not yet been implemented in Poland.

## Conclusions

In small long-term care facilities (LTCFs), prospective infection surveillance is more valuable, because the prevalence data are limited in terms of variation, distribution, risk factor of infections and also risk of lost data in connection with, for example, the hospitalisation of residents.

Prospective surveillance may be targeted using a threshold of the Barthel scale of 15, as identified with a specificity/sensitivity analysis.

Target surveillance based on the Barthel scale may especially be used in countries with limited resources in infection control. Further research on the basis of a larger group of residents is needed in order to be able to discuss the possibility of targeted surveillance in LTCFs.

The identified infection rates in the incidence study in Polish LTCFs are similar to other reports.
